# Effects of exercise snacks on body composition in adults: a systematic review and meta-analysis based on evidence from bioimpedance and DXA methods

**DOI:** 10.3389/fspor.2025.1679563

**Published:** 2026-01-08

**Authors:** Ruting Lin, Hongxian Zheng, Zhenyu Shuai, Mingkuai Wang, Xuanhui Guo, Yubo Wang, Wenqiang Wu

**Affiliations:** 1 College of Education, Beijing Sport University, Beijing, China; 2 Physical Education Department, College of Basic Education, Beijing College of Finance and Commerce, Beijing, China; 3 Cardiac Rehabilitation Center, Fuwai Hospital, Beijing, China

**Keywords:** exercise snack, body composition, meta-analysis, Obesity, body fat

## Abstract

This systematic review and meta-analysis examined the effects of exercise snacks (ES) on body composition in adults. A comprehensive search of PubMed, Scopus, Web of Science, Embase, FMRS, CINAHL, and the Cochrane Library was conducted from inception to May 2025, supplemented by backward and forward citation tracking. Eight articles comprising nine studies met the inclusion criteria. Meta-analysis revealed that ES interventions produced a statistically significant improvement in lean mass (MD = 0.52 kg, 95% CI: 0.13–0.91, *p* = 0.01). In contrast, no statistically significant effects were observed for body fat (MD = −0.04%, 95% CI: −0.73–0.65, *p* = 0.91) or body mass (MD = 0.03 kg, 95% CI: −0.53–0.60, *p* = 0.91). The findings support that ES interventions can enhance lean body mass. However, there is currently no convincing evidence that ES reduces body fat. The review followed PRISMA guidelines and was registered on PROSPERO (CRD420250651818).

**Systematic Review Registration:**

identifier CRD420250651818

## 1 Introduction

Non-communicable diseases (NCDs) remain the leading cause of death and illness worldwide, and addressing modifiable risk factors—such as tobacco use, excessive alcohol consumption, poor nutrition, and physical inactivity—presents a cost-effective strategy to mitigate the burden of both NCDs and their associated mental health impacts (1). Among these risk factors, physical inactivity stands out as a particularly significant contributor to a wide range of NCDs, including stroke, hypertension, type 2 diabetes, coronary heart disease, various cancers, dementia, depression, and premature mortality (2).

Beyond these conditions, physical inactivity also plays a crucial role in the development of unfavorable body composition, particularly through increased body fat accumulation and the loss of lean muscle mass (3). These changes in body composition are closely associated with a heightened risk of chronic diseases, further exacerbating the global health crisis. In 2022, nearly one-third of the global adult population (31.3%, or 1.8 billion people) was insufficiently active, an increase from 23.4% (900 million people) in 2000. Alarmingly, trends of rising physical inactivity were observed in nearly half of all countries and two-thirds of global regions (4). Traditional exercise models, however, face numerous barriers, such as the need for specific environments, specialized equipment, and significant time commitments, making it challenging for individuals to begin and sustain long-term physical activity (5).

In recent years, exercise snacks (ES) has gained attention as a flexible and sustainable physical activity strategy that may help overcome these barriers (6). Several meta-analyses have examined the effects of ES on body composition and provided preliminary evidence; for example, Wan et al. reported ES had no meaningful effects on body fat or body weight (7), whereas Rodríguez et al. similarly found no statistically significant impact of ES on body fat (8). However, a recent consensus statement provided a more stringent and specific definition of ES: it refers to short bouts of any type or intensity of exercise lasting ≤10 min (including multiple sets of interval training), performed multiple times (≥2 sessions/day) per day, with recovery periods of complete rest or at least 30 min between bouts (9). Existing evidence suggests that ES holds meaningful potential for improving public health, as it has been shown to increase muscular fitness via repeated neuromuscular activation and stimulation of muscle protein synthesis, while also interrupting sedentary behaviors with brief high-intensity interventions and promoting increased fat oxidation, thereby improving overall energy metabolism (10). Because many previous reviews do not fully conform to this new definition, the present study applied these stricter criteria within the updated conceptual framework to provide a more accurate evaluation of the impact of ES on body composition.

Although ES has demonstrated positive effects on health, evidence regarding its impact on body composition (such as body fat, lean mass, and body weight) remains inconsistent. For example, Wong et al. reported no significant changes in body composition after a 6-week ES intervention (11), whereas Zhou et al. observed a notable reduction in body weight following a 12-week program (12). Therefore, this study uses a systematic review and meta-analysis to assess the specific effects of ES on adult body composition. While previous meta-analyses have provided preliminary evidence regarding the effects of exercise snacks on body composition (7), the present meta-analysis strictly adhered to standardized inclusion criteria for ES, thereby offering new insights into the effect of ES on body composition.

## 2 Methods

This systematic review and meta-analysis were performed according to the Preferred Reporting Items for Systematic Reviews and Meta-Analysis (PRISMA) guideline (Supplementary Table S1) and registered with PROSPERO (CRD420250651818).

### 2.1 Eligibility criteria

To be included in this systematic review, previous studies must meet the following eligibility criteria in accordance with PICOS (Participants, Intervention, Comparator/Control, Outcomes, Study design).

Participants: the participants were adults with a mean age of ≥18 years

Intervention: Short bouts of exercise (ranging from 15 s to 10 min), performed at least twice a day, with intervals of 30 min or more.

Comparator/Control: No specific intervention, maintaining usual daily habits or receiving physical activity education (e.g., habitual exercise, usual physical activity, normal habitual behavior).

Outcomes: The outcomes include at least one measure related to body composition (e.g., Body mass, Lean mass, Body fat, etc.).

Study design: the design of the study was a randomized crossover or parallel trial.

Articles were excluded if they fulfilled the following criteria:1) Studies involving animal trials; 2) lacking accessible outcome data; 3) review papers and conference articles; and 4) repeated publications; 5）Studies including populations aged <18 years.

### 2.2 Data sources and search strategies

We searched PubMed, Scopus, Web of Science, Embase, FMRS, CINAHL, and the Cochrane Library. Databases for published studies on ES interventions and related topics. The search strategies were referenced from previous studies and keywords as follows (13): “Exercise snack”, “Snacktivity”, “Movement snack”, “Physical activity break”, “Movement break”, “Short bouts of exercise”, “Physical activity short bouts”, “Exercise short bouts”, “Short bouts of stair climbing”, “exercise”, “physical activity”, “movement”, “short bouts”, “brief bouts”, “intermittent bouts”, “low-volume bouts” and “mini bouts”. The search strategy is detailed in Supplementary Table S2. The databases were searched from inception to Oct 2025. In addition, review the bibliography of published review articles to find other studies that meet the inclusion criteria (7, 9, 13–18).

The retrieved records were manually deduplicated by an independent reviewer (H.Z.) using EndNote 20 (Clarivate Analytics, Philadelphia, Pennsylvania, USA). The deduplicated literature was then exported and provided to two independent researchers (H.Z. and R.L.), who screened the titles and abstracts according to the pre-set inclusion and exclusion criteria. If no consensus could be reached, a third independent researcher (Y.W.) reviewed the articles to determine whether they met the inclusion criteria. The two independent researchers (H.Z. and R.L.) reviewed the full text of the selected articles to determine the articles to be included. In cases where discrepancies or disagreements arose between the two authors, a third author (Y.W.) was consulted to facilitate resolution and reach a consensus. In addition, potential sources of relevant articles included references to previous systematic reviews on the topic and the expertise of the research team, who found some articles that might meet the inclusion criteria of this review but were not initially retrieved by literature.

### 2.3 Data extraction and outcomes

The process of data extraction was conducted according to the Cochrane Collaboration Handbook. Two authors (H.Z. and R.L.) independently performed data extraction, and when a decision disagreement happened, it was discussed with the third author (Y.W.) until a consensus was achieved. The extracted information from the publications included: the study (e.g., authors, year), participants (e.g., sample size, age, height, weight, sex, and eligibility criteria), grouping and sample size, ES interventions (e.g., frequency, intensity, time, type, duration), and outcome measures (19).

Most studies only report data for pre- and post-intervention. Thus, average change was calculated as the difference between the mean of data pre- and post-intervention. First, the difference in means was transformed via the following formula:

$$\mathrm{Mea}n_{\mathrm{change}}=\mathrm{Mea}n_{\mathrm{post}}-\mathrm{Mea}n_{\mathrm{pre}}$$

Where Mean_change_ is the difference in means, Mean_pre_ is the pre-intervention mean, and Mean_post_ is the post-intervention mean. Second, the standard deviation (SD) was calculated using the following formula based on the principles of the Cochrane Handbook for Systematic Reviews of Interventions:

$$SD_{\mathrm{change}}=\sqrt{{\mathrm{SD}}_{\mathrm{pre}}^{2}+{\mathrm{SD}}_{\mathrm{post}}^{2}-2\times \mathrm{Corr}\times SD_{\mathrm{pre}}\times SD_{\mathrm{post}}}$$

Where SD_change_ is the SD of the difference in means, SD_pre_ is the pre-intervention standard deviation, SD_post_ is the post-intervention standard deviation, Corr is the Pearson correlation coefficient, and following the recommendation of the Cochran handbook (19), we experiment with Corr = 0.80:

$$\mathrm{Corr}\;=\frac{\left(SD_{\mathrm{baseline}}^{2}+2SD_{\mathrm{final}}^{2}-SD_{\mathrm{change}}^{2}\right)}{2\times SD_{\mathrm{baseline}}\;\times SD_{\mathrm{final}}}$$

We referred to previous meta-analyses with similar results, with Corr values of 0.80 (20), 0.85 (21), and 0.89 (22), respectively. After sensitivity analyses, we ultimately chose Corr = 0.85. Moreover, if the original study reported standard error (SE), the SD can be obtained from the SE of a mean by multiplying by the square root of the sample size:

$$\mathrm{SD}=\mathrm{SE}\times \sqrt{N}$$

where *N* is the sample size.

### 2.4 Quality assessment

Two reviewers independently assessed the risk of bias, resolving disagreements through discussion when possible or, if necessary, by arbitration from a third researcher, using the Cochrane Collaboration's Risk of Bias Tool 2 (ROB 2) (23). This tool evaluates five domains: random sequence generation, random allocation concealment, blinding of outcome assessment, incomplete outcome data, and selective outcome reporting. For crossover trials, an additional domain assessing potential bias due to period and carryover effects was included. Additionally, the certainty of evidence for outcomes was evaluated using the Grading of Recommendations Assessment, Development, and Evaluation (GRADE) (24, 25). The quality of the GRADE evidence was graded as high, moderate, low, and very low based on the risk of bias/study limitations, inconsistency, indirectness, imprecision, and publication bias.

### 2.5 Statistical analysis

The mean difference (MD) was applied for variables measured on the same scale across all studies (lean mass, body fat, body mass). And MD was estimated using a random effects model. Meta-analysis was performed in Stata v15.1 (STATA Corp, College Station, TX) using the inverse-variance method. The *I*^2^ statistic was used to evaluate heterogeneity among the trials with the following criteria: trivial (<25%), low (25%–50%), moderate (50%–75%), and high (>75%) (26). Subgroup analysis was used to explore potential sources of heterogeneity (27). The Funnel plots and Egger tests were used to evaluate publication bias. If potential publication bias was detected, we used the trim and fill method for the sensitivity analysis of the results (28). All the statistical significance was set at a *p*-value of <0.05.

## 3 Results

### 3.1 Study selection

**Figure 1:**
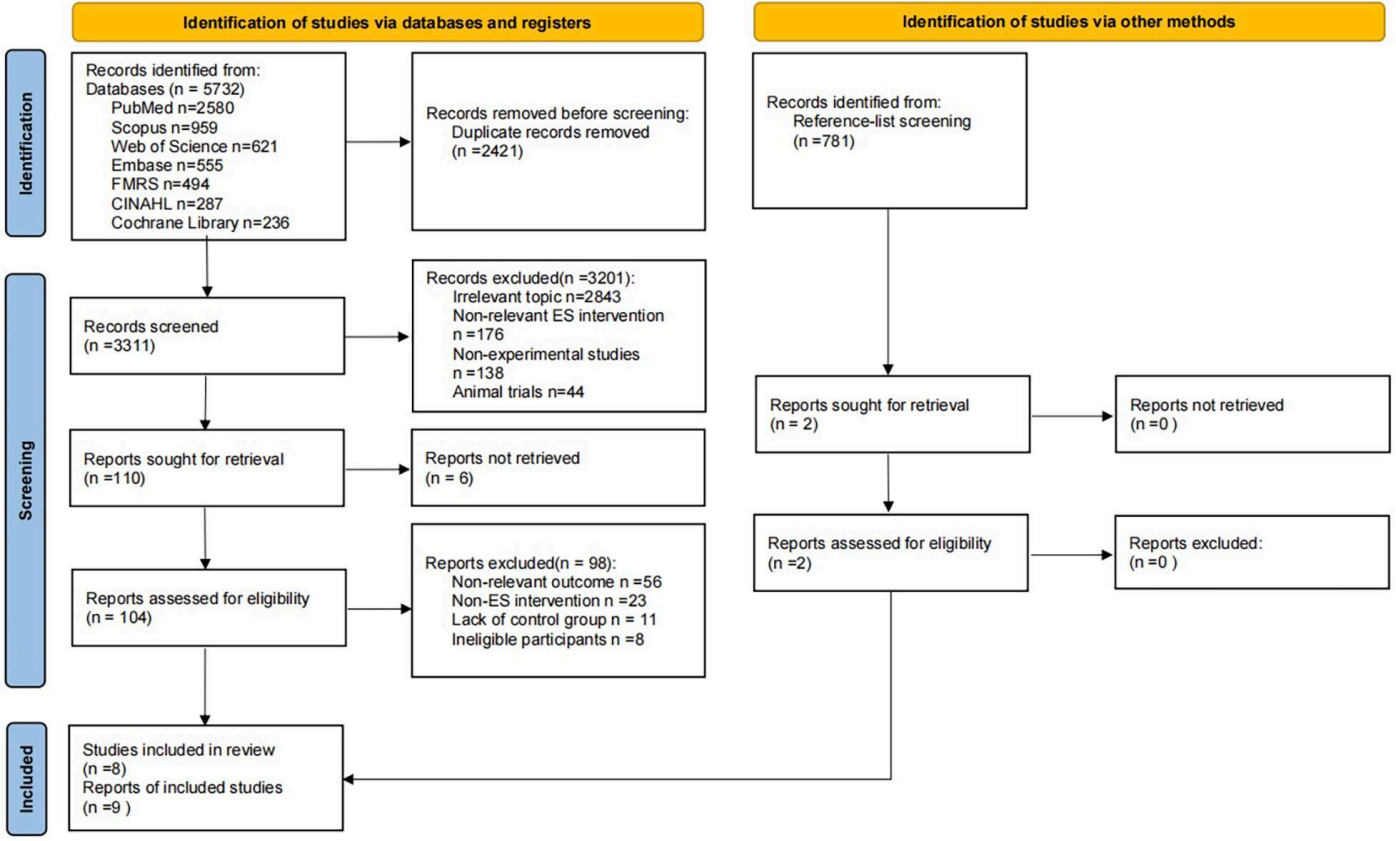
Study flowchart.

The screening process is detailed in Figure 1. This study retrieved 5,732 potentially relevant articles from PubMed (*n* = 2,580), Scopus (*n* = 959), Web of Science (*n* = 621), Embase (*n* = 555), FMRS (*n* = 494), CINAHL (*n* = 287), and Cochrane Library (*n* = 236). After removing 2,421 duplicate records, 3,201 records were excluded based on title and abstract. The remaining 110 publications underwent full-text review; of these, 6 were unavailable, and 98 were excluded based on the following criteria: irrelevant outcome measures (*n* = 56), interventions involving non-ES snacks (*n* = 23), lack of control groups (*n* = 11), and ineligible participants (*n* = 8). Additionally, by reviewing the reference lists of published review articles, we identified two more studies that met the inclusion criteria. Consequently, eight publications encompassing nine studies (seven randomized controlled trials and two crossover studies) were included in the systematic review and further analyzed through quantitative synthesis (12, 29–35).

### 3.2 Characteristics of included studies

#### 3.2.1 Participant characteristics

A total of 292 participants, with mean ages ranging from 18 to 74 years, were included. Based on the age criterion of 65 years, 272 were classified as adults (sedentary healthy individuals = 183, sedentary unhealthy individuals = 27, obese individuals = 62), and 20 were classified as older adults (healthy individuals = 20). The detailed characteristics of the included studies can be found in Table 1.

**Table 1:** Characteristics of the included studies (*n*  =  9).

N.	Study	Study type	Participants *N*, M/F	Age; BMI	Eligibility criteria	Exercise snack group (frequency, intensity, time, type)	Adherence	Control group	Outcome
1	Perkin et al., (29)	RCT	*N* = 20, 6/14	72 ± 5 years;	Community-dwelling older adults who were physically inactive but otherwise healthy.	F: 2/day for 28 days	98%, completing 55 out of 56 sessions.	Usual activity	Lean mass →
				26 ± 3 kg/m^2^		I: As many as possible			Body mass →
						T: 10 min (1 min on/1 min off)			Body fat % →
						T: 5 bodyweight exercises			
						Duration: 4 weeks			
						Other: with yoghurt supplementation			
2	Kennedy et al., (32)	RCT	*N* = 45, 22/23	42.3 ± 9 years; 25.5 ± 3.2 kg/m^2^	Sedentary but otherwise healthy adults.	F: 1–3 sessions/day, 5 days/week	Six participants of the stair climbing group dropped out of the study.	Not applicable	Body mass →
						I: 75 steps/min			Body fatness →
3	Mair et al., (33) (Exp.1)	crossover study	*N* = 31, 12/19	55–64 years; 27.7 ± 3.73 kg/m^2^	Sedentary but otherwise healthy adults.	T: 2 min/sprint	Adherence was 96%.	Habitual exercise	Body mass →
						T: stair climb			Body fat → Lean mass →
						Duration: 8 weeks			
						T: bench stepping			
						Duration: 4 weeks			
4	Mair et al., (33) (Exp.2)	crossover study	*N* = 31, 12/19	55–64 years; 25 ± 3.19 kg/m^2^	Sedentary but otherwise healthy adults.	F: 2–3 sessions/day, 2–3 days/week	Adherence was 96%.	Habitual exercise	Body mass →
						I: 75% HR			
									Body fat →
									Lean mass →
5	Brandt et al., (30)	RCT	*N* = 30,0/30	42.1 ± 11.1 years; 26.9 ± 4.3 kg/m^2^	Female employees with sedentary occupations; Engaged in resistance training less than 2 days/week prior to the study.	F: 5 days/week	Three participants of the IG dropped out of the study.	Usual activity	Muscle mass → Body fat ↓
						I: as many as possible			
						T: 10 minutes per session			
						T: Resistance training (RT)			
						Duration: 12 weeks			
6	Zhou & Gao et al., (12)	RCT	*N* = 27,13/14	22.14 ± 1.88years;	Sedentary but otherwise healthy adults.	F: 6/day, 4 days/week	All participants completed each intervention.	Not applicable	Weight →
				≧25 kg/m^2^		I: make an all-out effort			Fat% ↓
									Total fat mass ↓
						T: 22.7 ± 9.8s			
						T: stair climb sprint			
						Duration:12 weeks			
7	Chung et al. (31)	RCT	*N* = 36, 0/36	Middle-aged; ≧30% ≧30%	Middle-aged women with a body fat percentage of 30% or higher.	F: 3 sessions/day, 3 days/week	11 participants dropped out for medical or personal reasons.	No exercise, only 300 kcal calorie restriction	Weight →
									Lean mass →
						I: 4 h between sessions.			Fat mass →
						T: 10 minper session			Fat percentage →

						T: treadmill exercise			
						Duration: 12 weeks			
8	Babir et al. (35)	RCT	*N* = 77, 21/56	EXP: 53 ± 6years; 27.5 ± 2.7 kg/m^2^ CON: 54 ± 7years; 26.9 ± 2.7 kg/m^2^	Healthy adults but physically inactive.	F: 3 sessions/day, 3 days/week	Adherence of 97%.	Stretching exercises	Body mass →
						I: relatively lower intensity			
						T: 30–60s/bouts			
						T: bodyweight exercises			
						Duration: 12 weeks			
9	Yun et al., (34)	RCT	*N* = 26,0/26	25.4 ± 4.9 years; 25.3 ± 1.8 kg/m2	Women with overweight or obesity.	F: 3 sessions/day, 5 days/week	Of the 26 participants randomized, 25 completed their assigned treatment group.	Current leisure time activity	Weight ↓
						I: as quickly as possible, one step at a time			
						T: 20 s/bouts			
						T: stair climbing exercises			
						Duration: 4 weeks			

#### 3.2.2 Es protocol

In the included eight publications and nine studies, there is no widely accepted consensus on ES intervention protocols. The included studies employed various types of ES, categorized into bodyweight exercises (*n* = 2) (29, 35); stair climb (*n* = 3) (12, 32, 34); bench stepping (*n* = 1) (33); Resistance training (*n* = 1) (30); treadmill exercise (*n* = 1) (31).

The time of each ES session was classified as follows: 20 s to less than three minutes per session (12, 32–35) 10 min per session (30, 31) and 10 min (one min on/one min off) per session (29).

Intensity standards were not determined using standardized measurement methods (e.g., HR or RPE). Among them, one study require participants to “go all out” (12), three studies ask subjects to exercise as fast as possible or repeat as many times as possible (29, 30, 34), one study asked for comfortable but brisk rate exercise (32), one study required high-intensity (35), one study sets the high-intensity target at the level corresponding to 75% of heart rate reserve (33), while in the other one studies, there are no specific requirements for the exercise intensity (31).

The duration of ES interventions varied across the included studies, with three studies implementing 4-week protocols (29, 33, 34), one studies implementing 8-week protocols (32), and four studies implementing 12-week protocols (12, 30, 31, 35).

The frequency of ES interventions was examined in terms of daily and weekly occurrences. Daily intervention frequency varied among studies, with one study implementing interventions once per day (30), one study implementing them twice per day (29), five studies implementing them three times per day (31–35), and one studies implementing them six per day (12).

Weekly frequency was inconsistently reported, with three studies implemented interventions three days per week, one study implementing interventions four days per week (12), three studies implementing interventions five days per week (30, 32, 34), and one study seven days per week (29).

#### 3.2.3 Outcome measurements

The included studies examined the effects of ES interventions on body fat, lean mass, and body mass in participants. For body fat, three studies used dual-energy x-ray absorptiometry (DXA) (QDR software version 12.4.2, Hologic Discovery W, Bedford, MA; Lunar iDXA, GE Healthcare, Madison, WI; DEXA, Discovery Wi, USA) (12, 29); three studies used bioelectrical impedance analysis (BIA) using Bodystat® 1,500 (32), InBody 720 (31) and SECA® mBCA 515 (30).

For lean mass, two studies used DXA (29, 33); two studies used BIA (31, 34); One study used SECA® mBCA 515 scales (30).

For body mass, two studies used electronic scales, the BC543 scale (Tanita, Amsterdam, Netherlands) (29), and the Body Composition Analyzer (BC-418MA, Tanita Corporation, Tokyo, Japan) (33); One study employed DXA (12); three studies employed BIA (31, 32, 34); One study did not report on the instruments used to measure body weight (35).

#### 3.2.4 Adherence of Es interventions

In terms of adherence, one study demonstrated adherence rates of 100%, all participants completed each intervention and test without any adverse events (12). Four studies showed adherence rates between 90% and 100% (29, 33–35). One study reported adherence rates between 80% and 90% (30). Additionally, two studies revealed adherence rates between 70% and 80% (31, 32).

### 3.3 Quality assessment

**Figure 2:**
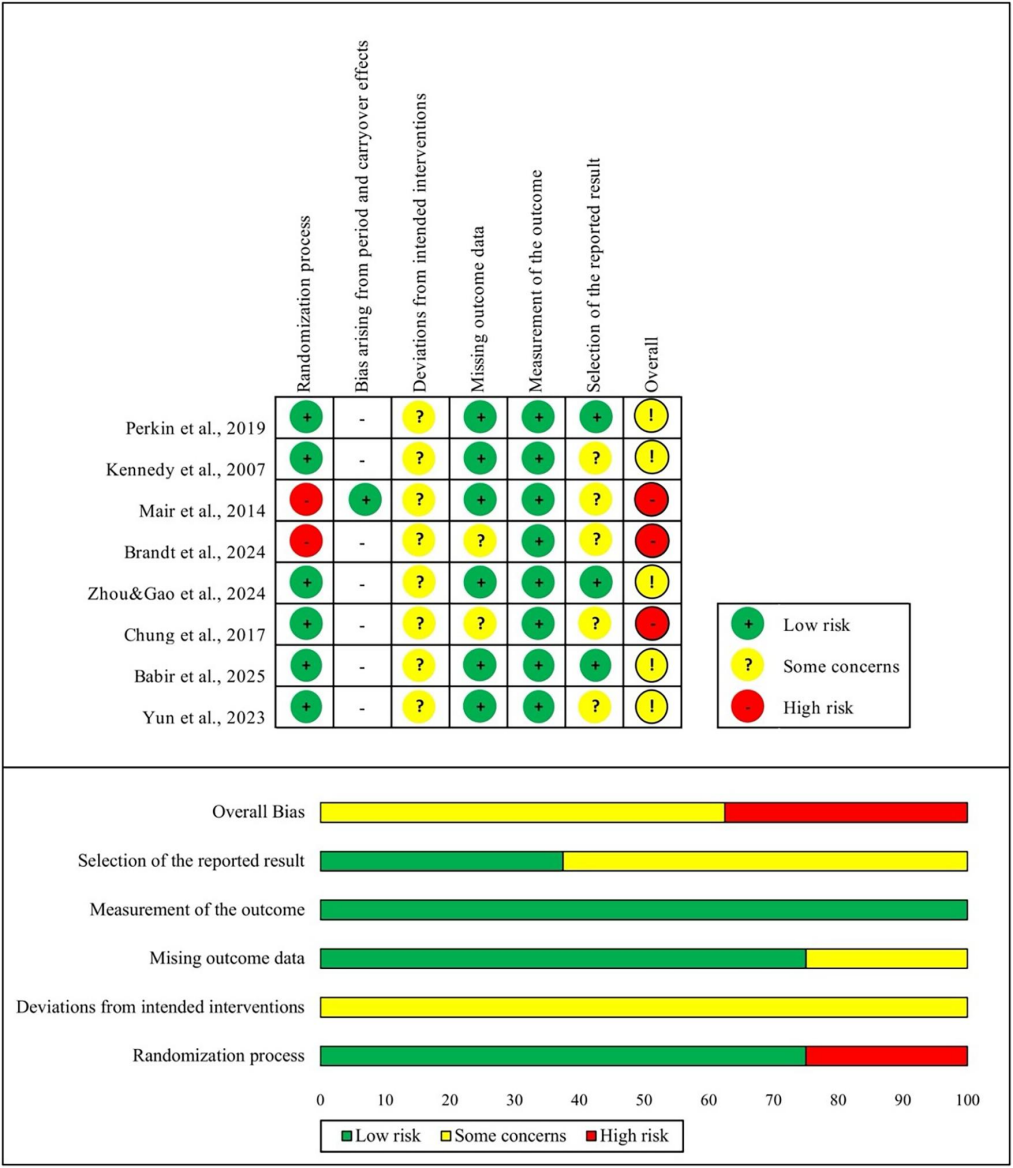
Risk of bias in the included studies.

The risk of bias assessment (Figure 2) for the eight included publications showed that six studies were rated as low risk for the randomization process (12, 29, 31, 32, 34, 35), whereas two studies were judged as high risk due to insufficient reporting and the absence of randomization procedures (30, 33). For deviations from intended interventions, all studies were rated as having some concerns, primarily because of limitations in blinding procedures (12, 29–35). In missing outcome data, two studies showed some concerns (30, 31), while the rest were low risk (12, 29, 32–35). All studies were low risk for outcome measurement (12, 29–35). For selection of the reported results, three studies were rated as having low risk (12, 29–35), whereas five studies were accessed as some concerns due to the absence of a registered trial protocol (12, 29–35). Additionally, since Mair et al. conducted a crossover trial, the evaluation criteria differed slightly; the study was assessed as low risk in the domain of bias arising from period and carryover effects. Overall, five studies exhibited some concerns related to risk of bias, while three studies were identified as being at high risk. These findings suggest moderate to high methodological quality, with some limitations in reporting and protocol adherence in a subset of studies. Additionally, Figure 2 shows an overview of risk of bias across all included studies. The quality of evidence for outcomes was evaluated as low to moderate, and details for the evaluation of the GRADE framework are presented in Supplementary Table S3.

### 3.4 Meta-analysis

A subgroup analysis was conducted to explore potential sources of heterogeneity, considering two main factors: frequency (i.e., less than three times per day, three or more times per day) and duration (i.e., less than 12 weeks, 12 weeks or longer).

According to World Health Organization's guidelines (WHO, 2020), adults should participate in at least 150 min of moderate-intensity aerobic exercise per week, with a minimum of 30 min per day for at least five days. Exercise snacks, an efficient strategy involving brief sessions of about 10 minutes, should be performed at least three times daily. Based on the intervention designs in the included studies, exercise snacks frequency was categorized as less than three times, three or more times per day, with an intervention duration of 12 weeks.

#### 3.4.1 Effects of Es on lean mass

**Figure 3:**
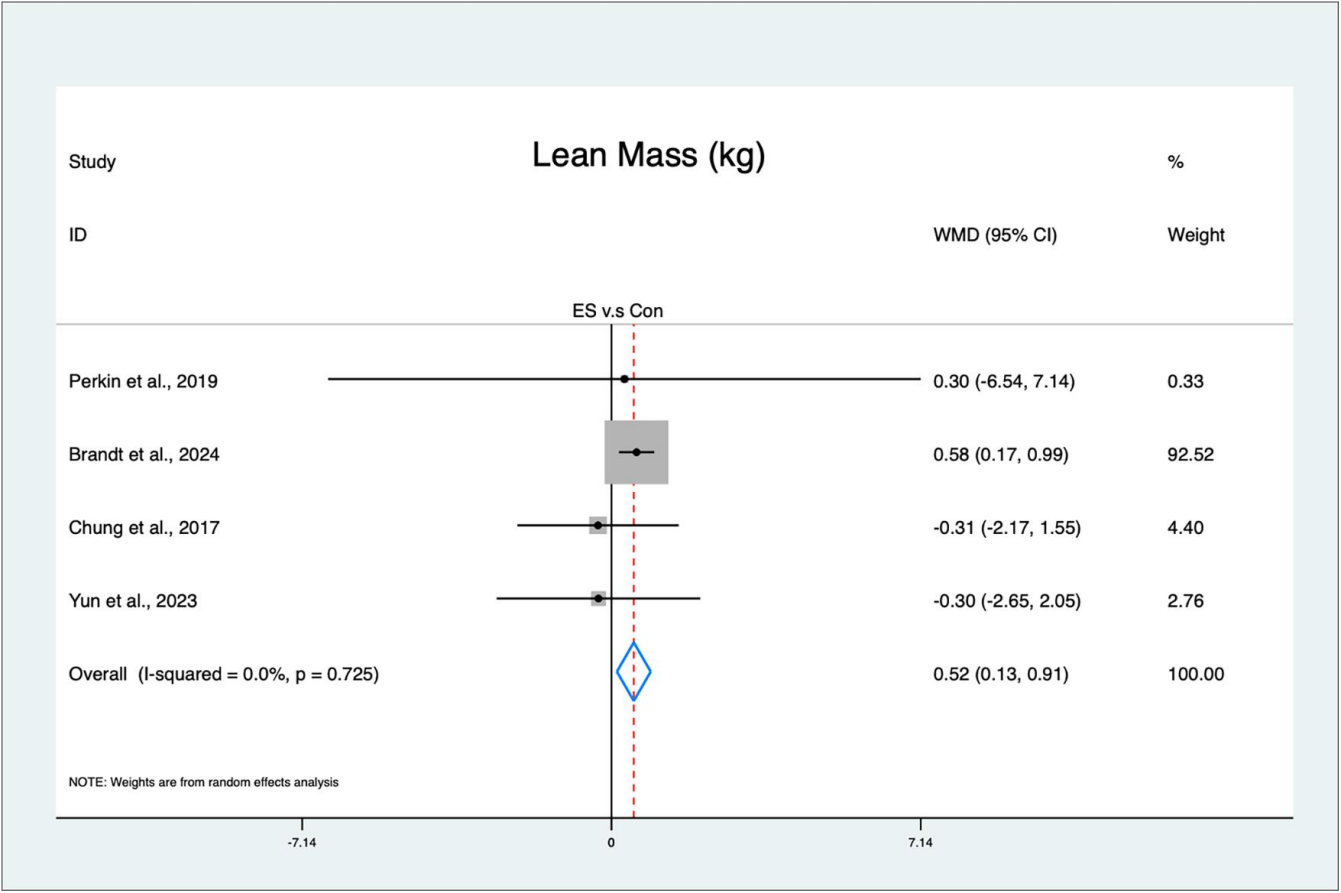
Forest plot of the effects of ES on lean mass.

The meta-analysis indicates that the ES interventions statistically significantly improved lean mass (MD = 0.52 kg, 95% CI: 0.13–0.91, *p* = 0.01, low GRADE, Figure 3), with low heterogeneity (*I*^2^ = 0%, *p* = 0.73). The funnel plot (Supplementary Figure S3) and Egger's test (*t* = −1.82, *p* = 0.21) indicated no publication bias.

#### 3.4.2 Effects of Es on body fat

**Figure 4:**
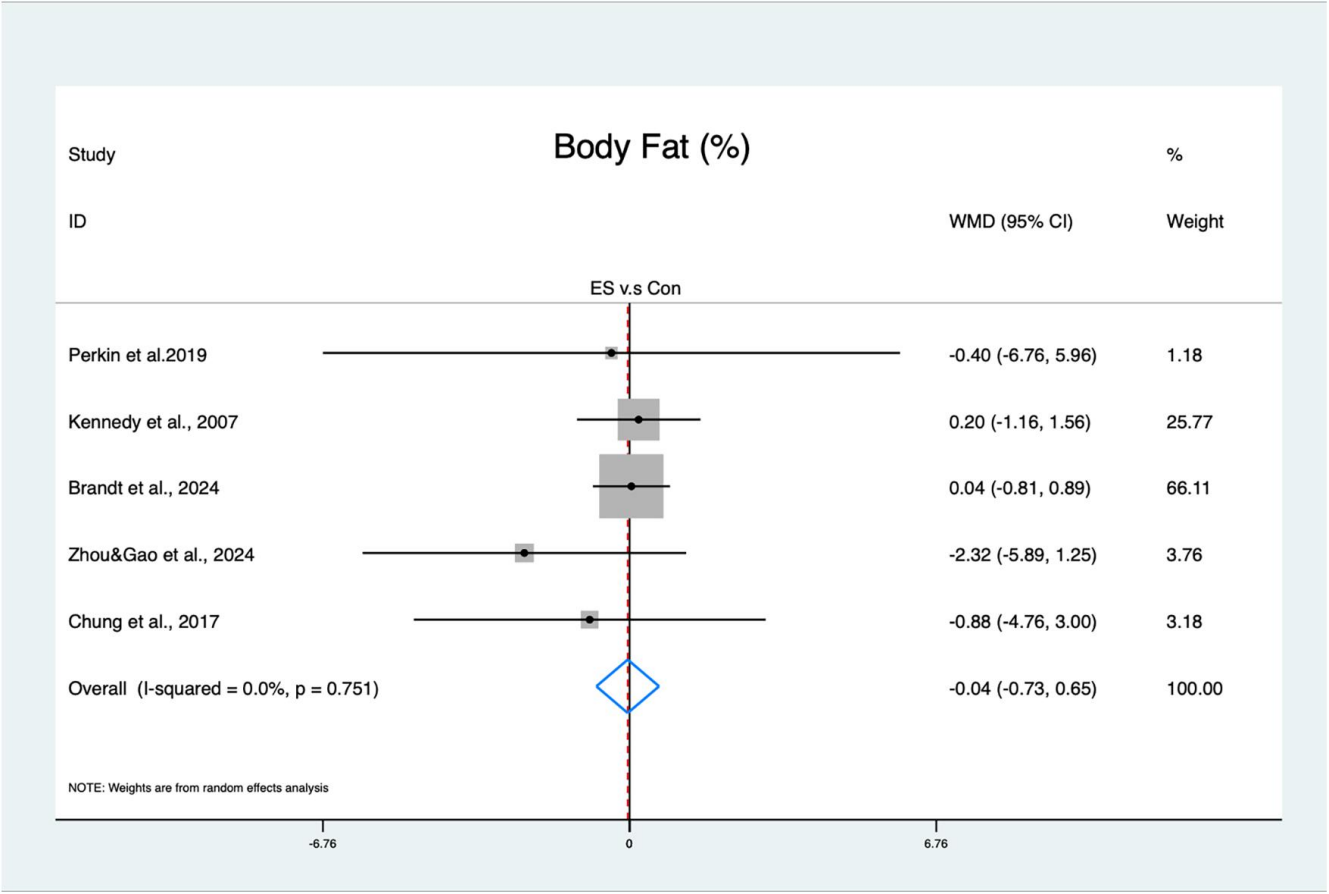
Forest plot of the effects of ES on body fat.

For body fat, the ES interventions showed a statistically non-significant effect (MD = −0.04%, 95% CI: −0.73–0.65, *p* = 0.91, moderate GRADE, Figure 4), with no heterogeneity (*I*^2^ = 0%, *p* = 0.75). The funnel plot (Supplementary Figure S1) and Egger's test (*t* = −1.61, *p* = 0.21) indicated no publication bias.

#### 3.4.3 Effects of Es on body mass

**Figure 5:**
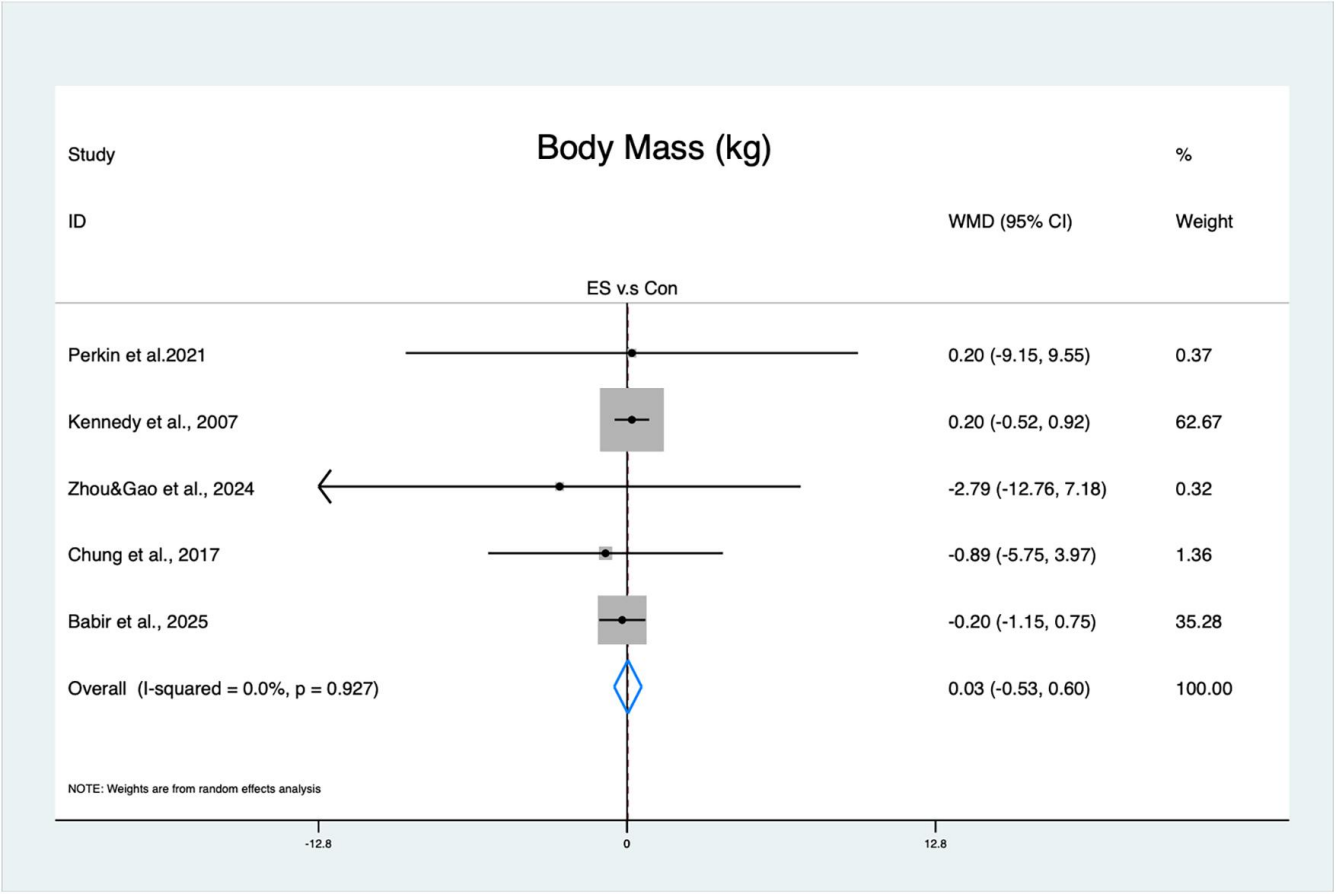
Forest plot of the effects of ES on body mass.

For body mass, the result was not statistically significant (MD = 0.03 kg, 95% CI: −0.53–0.60, *p* = 0.91, moderate GRADE, Figure 5), with no heterogeneity (*I*^2^ = 0%, *p* = 0.93). The funnel plot (Supplementary Figure S2) and Egger's test (t = −1.42, *p* = 0.25) indicated no publication bias.

## 4 Discussion

This systematic review and meta-analysis evaluated the effects of ES on body composition in adults. The results indicated that ES interventions statistically significant increased lean body mass, whereas no statistically significant overall effects were observed for body fat or body mass, with adherence to the ES interventions generally high across the included studies.

The meta-analysis suggests that ES had statistically significant impact on lean mass. However, findings across studies remain inconsistent. For example, Perkin et al. found that a 4-week ES program reduced body fat while increasing leg lean mass by 1% and thigh muscle cross-sectional area (mCSA) by 2% (33). However, these findings did not reach statistical significance. Brandt et al. reported a significant increase in muscle mass [+0.42 (0.54) kg, or +2%] following a 12-week resistance-focused ES intervention (34). Such variability among studies may be attributed to differences in training design and inadequate standardization of essential training parameters, including frequency, rest intervals, and progression across training cycles (36). Skeletal muscle hypertrophy is a complex, multifactorial adaptive process in response to resistance exercise training (RET), predominantly driven by mechanical tension and the activation of mechanotransductive intracellular signaling cascades (e.g., via mechanotransduction-mediated pathways signaling through mTORC1) (37). In practical application, it is important to emphasize fundamental elements such as progressive training intensity, sufficient training volume, adequate recovery, and long-term adherence to effectively stimulate muscle hypertrophy. Therefore, future studies should provide detailed reporting of ES intervention variables and further investigate how variations in these parameters influence intervention effectiveness.

The meta-analysis showed that ES had no statistically significant impact on body fat in adults. However, findings across studies remain inconsistent. For example, Wong et al. reported no significant change in body fat after a 6-week ES intervention (11), while Kennedy et al. observed no improvements in body composition following 8 weeks of ES (32). In contrast, other studies support ES's potential to reduce body fat. Chung et al. reported a significant reduction in body fat after a 12-week intervention (31), and Zhou et al. observed decreased abdominal visceral fat following a similar protocol (12). Additionally, Zhang et al. demonstrated that adequate exercise dosage can enhance thermogenesis in adipose tissue, regulate endocrine pathways, and improve fat oxidation by influencing key metabolic organs, including skeletal muscle and liver (38). These discrepancies may stem from insufficient ES doses in studies that did not detect changes in body fat. For instance, Mair et al. implemented a 4-week stair-stepping program (2–3 sessions/day, 2–3 min/session, 3 days/week; total 12–27 min/week) (33), while Kennedy et al. used an 8-week stair-climbing protocol (1–3 sessions/day, 2 min/session, 5 days/week; total 10–30 min/week) (32). Both protocols fall well below the World Health Organization's recommendation of at least 150 min of moderate-intensity aerobic exercise per week and fail to meet the fat reduction threshold outlined in physical activity guidelines (39).

Regarding body mass, the results indicated that ES interventions may have limited efficacy in reducing body mass among adults. However, the outcomes varied across studies depending on participant characteristics, intervention design, and study methodology. For example, Kennedy et al. conducted an 8-week stair-climbing ES intervention in healthy adults and observed a slight increase in average body mass (32). In contrast, Zhou et al. applied a similar 12-week stair-climbing protocol in sedentary obese adults and reported a decrease in body mass, though the change was not statistically significant (12). These findings suggest that ES effects on body weight may vary across different populations. Current interventions may be more effective in overweight or obese individuals (12). However, body mass is influenced by both energy intake and expenditure, and most existing studies do not control for dietary factors (40). Only three of the included studies provided dietary information (30, 32, 33). Therefore, future research should incorporate dietary logs or control for caloric intake to better isolate the effects of ES. Combining ES with nutritional strategies may improve the effectiveness of body mass management interventions.

## 5 Limitations

There are some limitations to be considered in this systematic review. First, the included studies varied in intervention design, incorporating different types of exercise such as stair climbing, cycling, and sprinting. Additionally, the absence of uniform standards for intervention frequency, intensity, and duration results in inevitable bias. Future research should explore the effects of various exercise modalities and dosages and aim to establish standardized classifications for ES interventions. Second, most studies inadequately controlled for confounding factors such as dietary intake and habitual physical activity, which may have influenced the accuracy of the results. Future research should incorporate dietary logs and physical activity monitoring to improve variable control and more accurately evaluate the real-world effectiveness of ES interventions. Furthermore, the included studies varied in participant characteristics (e.g., BMI, sex, and age), and the certainty of evidence was rated as low for one outcome and moderate for two outcomes, indicating limited certainty regarding the observed effects of ES on these outcomes, which may change substantially with future research.

## 6 Conclusions

In conclusion, this systematic review and meta-analysis identified the relationship between ES and body composition in adults. The findings support that ES interventions can enhance lean body mass. However, there is currently no convincing evidence that ES reduces body fat. Future research is encouraged to investigate the effectiveness of ES across different populations, further clarify how variations in ES frequency and duration affect outcomes, and elucidate the physiological mechanisms through which ES influences body composition.

## Data Availability

The original contributions presented in the study are included in the article/Supplementary Material, further inquiries can be directed to the corresponding author.
