# Supplementary material for: Effects of exercise snacks on body composition in adults: a systematic review and meta-analysis based on evidence from bioimpedance and DXA methods
Source: Front Sports Act Living. 2026 Jan 8;7:1679563. doi: 10.3389/fspor.2025.1679563 (PMC12823816; doi:10.3389/fspor.2025.1679563)
Supplement: Supplementary file 1 [file Datasheet1.pdf]

Supplementary Material

1 PRISMA Guideline

Supplementary Table 1.

Section and Topic	Item #	Checklist item	Location where item is reported
**TITLE**			
Title	1	Identify the report as a systematic review.	Title
**ABSTRACT**			
Abstract	2	See the PRISMA 2020 for Abstracts checklist.	Abstract
**INTRODUCTION**			
Rationale	3	Describe the rationale for the review in the context of existing knowledge.	Introduction
Objectives	4	Provide an explicit statement of the objective(s) or question(s) the review addresses.	Introduction
**METHODS**			
Eligibility criteria	5	Specify the inclusion and exclusion criteria for the review and how studies were grouped for the syntheses.	Eligibility criteria
Information sources	6	Specify all databases, registers, websites, organisations, reference lists and other sources searched or consulted to identify studies. Specify the date when each source was last searched or consulted.	Data sources and search strategies
Search strategy	7	Present the full search strategies for all databases, registers and websites, including any filters and limits used.	Data sources and search strategies
Selection process	8	Specify the methods used to decide whether a study met the inclusion criteria of the review, including how many reviewers screened each record and each report retrieved, whether they worked independently, and if applicable, details of automation tools used in the process.	Data extraction and outcomes
Data collection process	9	Specify the methods used to collect data from reports, including how many reviewers collected data from each report, whether they worked independently, any processes for obtaining or confirming data from study investigators, and if applicable, details of automation tools used in the process.	Data extraction and outcomes
Data items	10a	List and define all outcomes for which data were sought. Specify whether all results that were compatible with each outcome domain in each study were sought (e.g. for all measures, time points, analyses), and if not, the methods used to decide which results to collect.	Eligibility criteria

	10b	List and define all other variables for which data were sought (e.g. participant and intervention characteristics, funding sources). Describe any assumptions made about any missing or unclear information.	Eligibility criteria
Study risk of bias assessment	11	Specify the methods used to assess risk of bias in the included studies, including details of the tool(s) used, how many reviewers assessed each study and whether they worked independently, and if applicable, details of automation tools used in the process.	Methods
Effect measures	12	Specify for each outcome the effect measure(s) (e.g. risk ratio, mean difference) used in the synthesis or presentation of results.	Methods
Synthesis methods	13a	Describe the processes used to decide which studies were eligible for each synthesis (e.g. tabulating the study intervention characteristics and comparing against the planned groups for each synthesis (item #5)).	Methods
	13b	Describe any methods used to tabulate or visually display results of individual studies and syntheses.	Methods
	13c	Describe any methods used to synthesize results and provide a rationale for the choice(s). If meta-analysis was performed, describe the model(s), method(s) to identify the presence and extent of statistical heterogeneity, and software package(s) used.	Methods
	13d	Describe any methods used to explore possible causes of heterogeneity among study results (e.g. subgroup analysis, meta-regression).	Methods
	13e	Describe any sensitivity analyses conducted to assess robustness of the synthesized results.	Methods
Reporting bias assessment	14	Describe any methods used to assess risk of bias due to missing results in a synthesis (arising from reporting biases).	Methods
Certainty assessment	15	Describe any methods used to assess certainty (or confidence) in the body of evidence for an outcome.	Methods
**RESULTS**			
Study selection	16a	Describe the results of the search and selection process, from the number of records identified in the search to the number of studies included in the review, ideally using a flow diagram.	Fig 1. and Study selection
	16b	Cite studies that might appear to meet the inclusion criteria, but which were excluded, and explain why they were excluded.	Fig 1. and Study selection
Study characteristics	17	Cite each included study and present its characteristics.	Table1. and Characteristics of included studies
Risk of bias in studies	18	Present assessments of risk of bias for each included study.	Fig 2. and Quality assessment
Results of individual studies	19	For all outcomes, present, for each study: (a) summary statistics for each group (where appropriate) and (b) an effect estimate and its precision (e.g. confidence/credible interval), ideally using structured tables or plots.	Fig 3-5.
Results of syntheses	20a	For each synthesis, briefly summaries the characteristics and risk of bias among contributing studies.	Characteristics of included studies and Meta-analysis
	20b	Present results of all statistical syntheses conducted. If meta-analysis was done, present for each the summary estimate and its precision (e.g. confidence/credible interval) and measures of statistical heterogeneity. If comparing groups, describe the direction of the effect.	Fig 3-5. and Meta-analysis

	20c	Present results of all investigations of possible causes of heterogeneity among study results.	Fig 3-5. and Meta-analysis
Reporting biases	21	Present assessments of risk of bias due to missing results (arising from reporting biases) for each synthesis assessed.	Meta-analysis
Certainty of evidence	22	Present assessments of certainty (or confidence) in the body of evidence for each outcome assessed.	Meta-analysis
**DISCUSSION**			
Discussion	23a	Provide a general interpretation of the results in the context of other evidence.	Discussion
	23b	Discuss any limitations of the evidence included in the review.	Discussion
	23c	Discuss any limitations of the review processes used.	Discussion
	23d	Discuss implications of the results for practice, policy, and future research.	Discussion
**OTHER INFORMATION**			
Registration and protocol	24	Provide registration information for the review, including register name and registration number, or state that the review was not registered.	Methods
Support	25	Describe sources of financial or non-financial support for the review, and the role of the funders or sponsors in the review.	Funding
Competing interests	26	Declare any competing interests of review authors.	Conflict of Interest
Availability of data, code and other materials	27	Report which of the following are publicly available and where they can be found template data collection forms; data extracted from included studies; data used for all analyses; analytic code; any other materials used in the review.	Data Availability Statement

From: Page MJ, McKenzie JE, Bossuyt PM, Boutron I, Hoffmann TC, Mulrow CD, et al. The PRISMA 2020 statement: an updated guideline for reporting systematic reviews. BMJ 2021;372:n71. doi: 10.1136/bmj.n71

2 Search strategy

Supplementary Table 2.

Database	Query	Results
PubMed	("Exercise snack*" OR "Snacktivity" OR "Movement snack*" OR "Physical activity break*" OR "Movement break*" OR "Short bouts of exercise" OR "Physical activity short bouts" OR "Exercise short bouts" OR "Short bouts of stair climbing")[tiab]) OR (("exercise" OR "physical activity" OR "movement")[tiab] AND ("short bouts" OR "brief bouts" OR "intermittent bouts" OR "low-volume bouts" OR "mini bouts")[tiab])	2580
Scopus	TITLE-ABS-KEY(("Exercise snack*" OR "Snacktivity" OR "Movement snack*" OR "Physical activity break*" OR "Movement break*" OR "Short bouts of exercise" OR "Physical activity short bouts" OR "Exercise short bouts" OR "Short bouts of stair climbing")) OR TITLE-ABS-KEY (("exercise" OR "physical activity" OR "movement") AND ("short bouts" OR "brief bouts" OR "intermittent bouts" OR "low-volume bouts" OR "mini bouts"))	959
Web of Science	TS=("Exercise snack*" OR "Snacktivity" OR "Movement snack*" OR "Physical activity break*" OR "Movement break*" OR "Short bouts of exercise" OR "Physical activity short bouts" OR "Exercise short bouts" OR "Short bouts of stair climbing") OR TS=(("exercise" OR "physical activity" OR "movement") AND ("short bouts" OR "brief bouts" OR "intermittent bouts" OR "low-volume bouts" OR "mini bouts"))	621
Embase	("Exercise snack*" OR "Snacktivity" OR "Movement snack*" OR "Physical activity break*" OR "Movement break*" OR "Short bouts of exercise" OR "Physical activity short bouts" OR "Exercise short bouts" OR "Short bouts of stair climbing") OR ("exercise" OR "physical activity" OR "movement") AND ("short bouts" OR "brief bouts" OR "intermittent bouts" OR "low-volume bouts" OR "mini bouts")	555
FMRS	("Exercise snack*"[TIAB] OR "Snacktivity"[TIAB] OR "Movement snack*"[TIAB] OR "Physical activity break*"[TIAB] OR "Movement break*"[TIAB] OR "Short bouts of exercise"[TIAB] OR "Physical activity short bouts"[TIAB] OR "Exercise short bouts"[TIAB] OR "Short bouts of stair climbing"[TIAB]) OR ("exercise"[TIAB] OR "physical activity"[TIAB] OR "movement"[TIAB]) AND ("short bouts"[TIAB] OR "brief bouts"[TIAB] OR "intermittent bouts"[TIAB] OR "low-volume bouts"[TIAB] OR "mini bouts"[TIAB])	494

CINAHL	XB ("Exercise snack*" OR "Snacktivity" OR "Movement snack*" OR "Physical activity break*" OR "Movement break*" OR "Short bouts of exercise" OR "Physical activity short bouts" OR "Exercise short bouts" OR "Short bouts of stair climbing") OR XB (("exercise" OR "physical activity" OR "movement") AND ("short bouts" OR "brief bouts" OR "intermittent bouts" OR "low-volume bouts" OR "mini bouts"))	287
Cochrane Library	("Exercise snack*" OR "Snacktivity" OR "Movement snack*" OR "Physical activity break*" OR "Movement break*" OR "Short bouts of exercise" OR "Physical activity short bouts" OR "Exercise short bouts" OR "Short bouts of stair climbing") in Title Abstract Keyword OR("exercise" OR "physical activity" OR "movement") AND ("short bouts" OR "brief bouts" OR "intermittent bouts" OR "low-volume bouts" OR "mini bouts") in Title Abstract Keyword	236

3 funnel plot

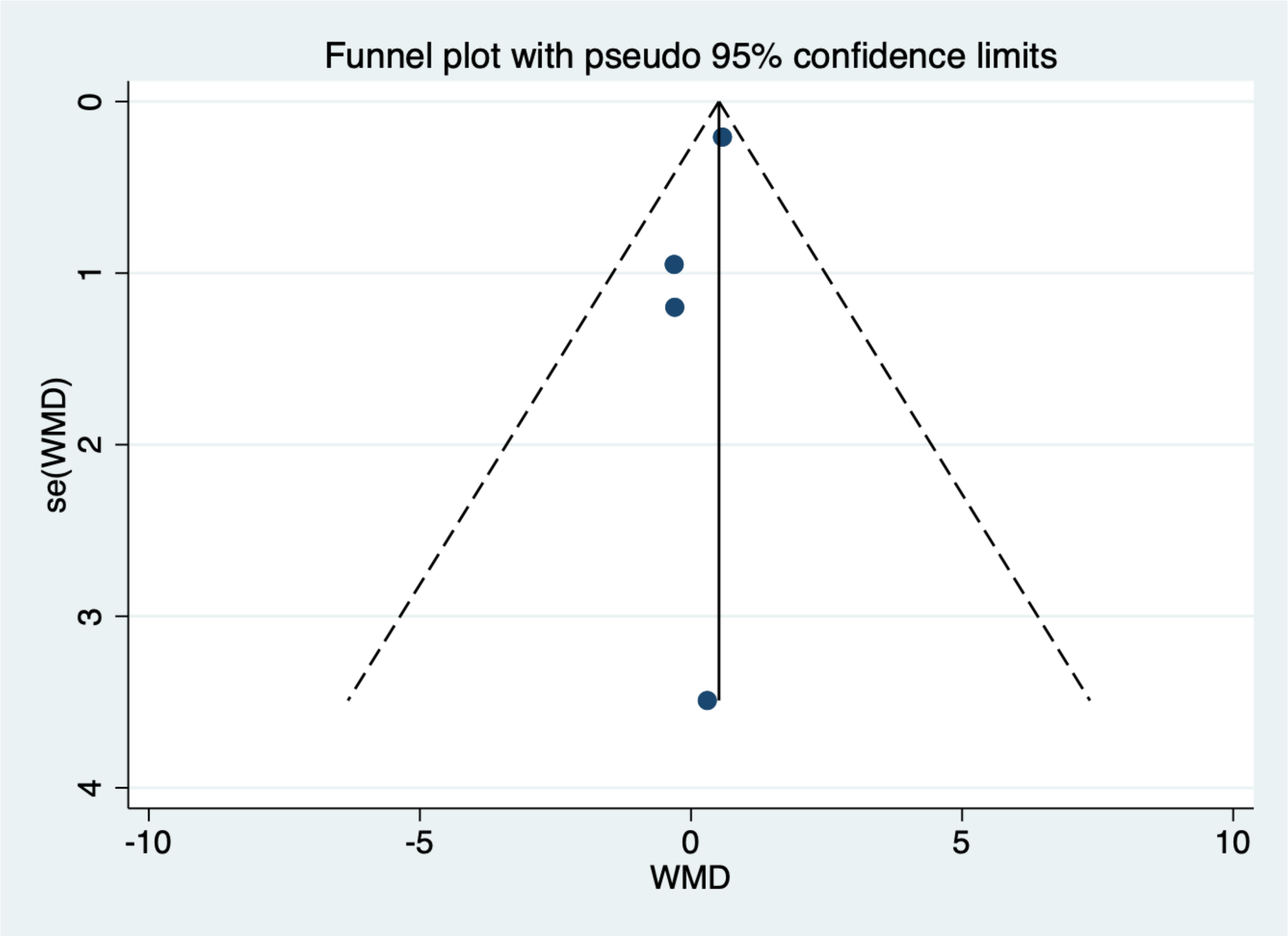

Supplementary Figure 1. The funnel plot of lean mass.

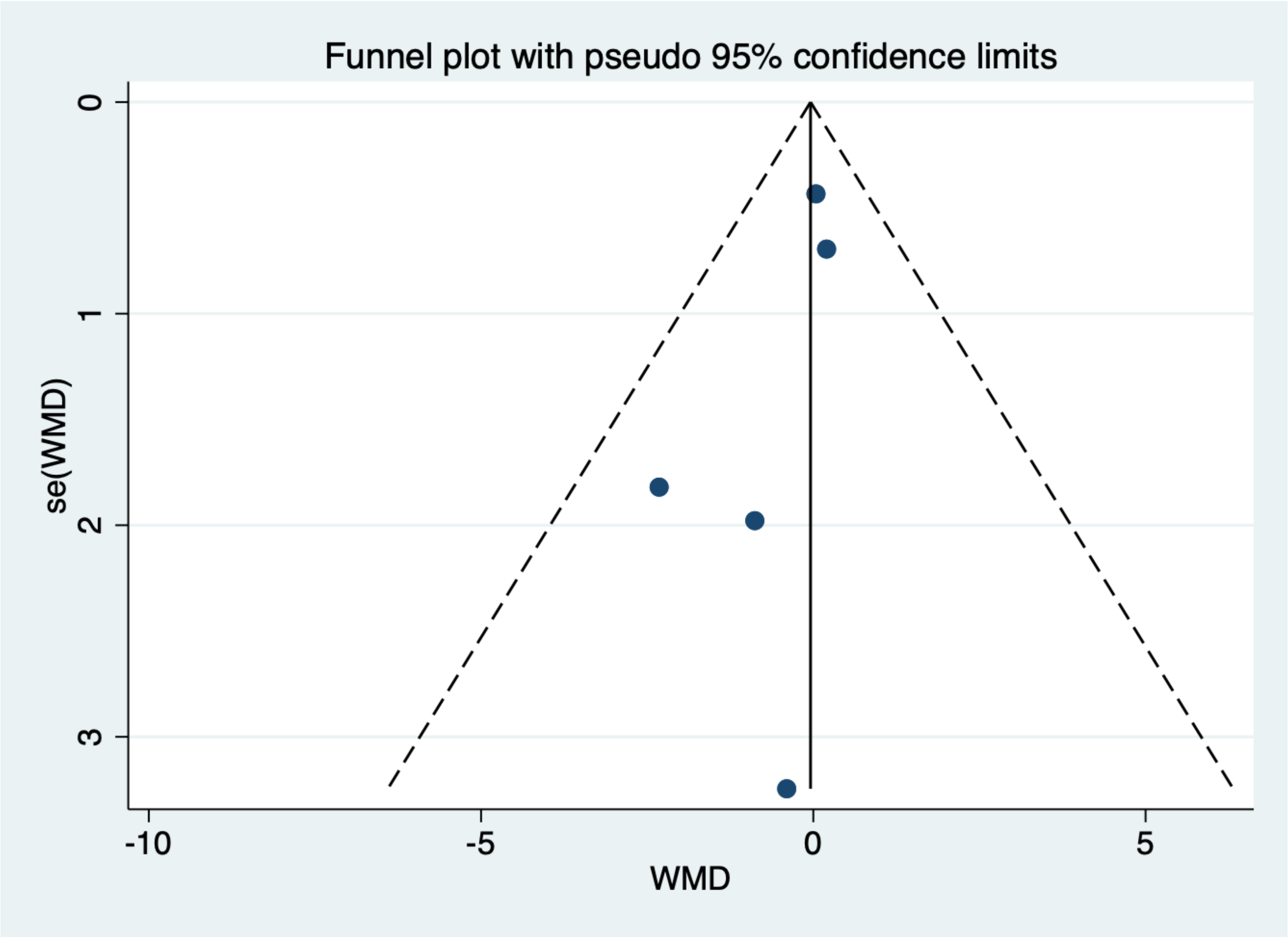

Supplementary Figure 2. The funnel plot of body fat.

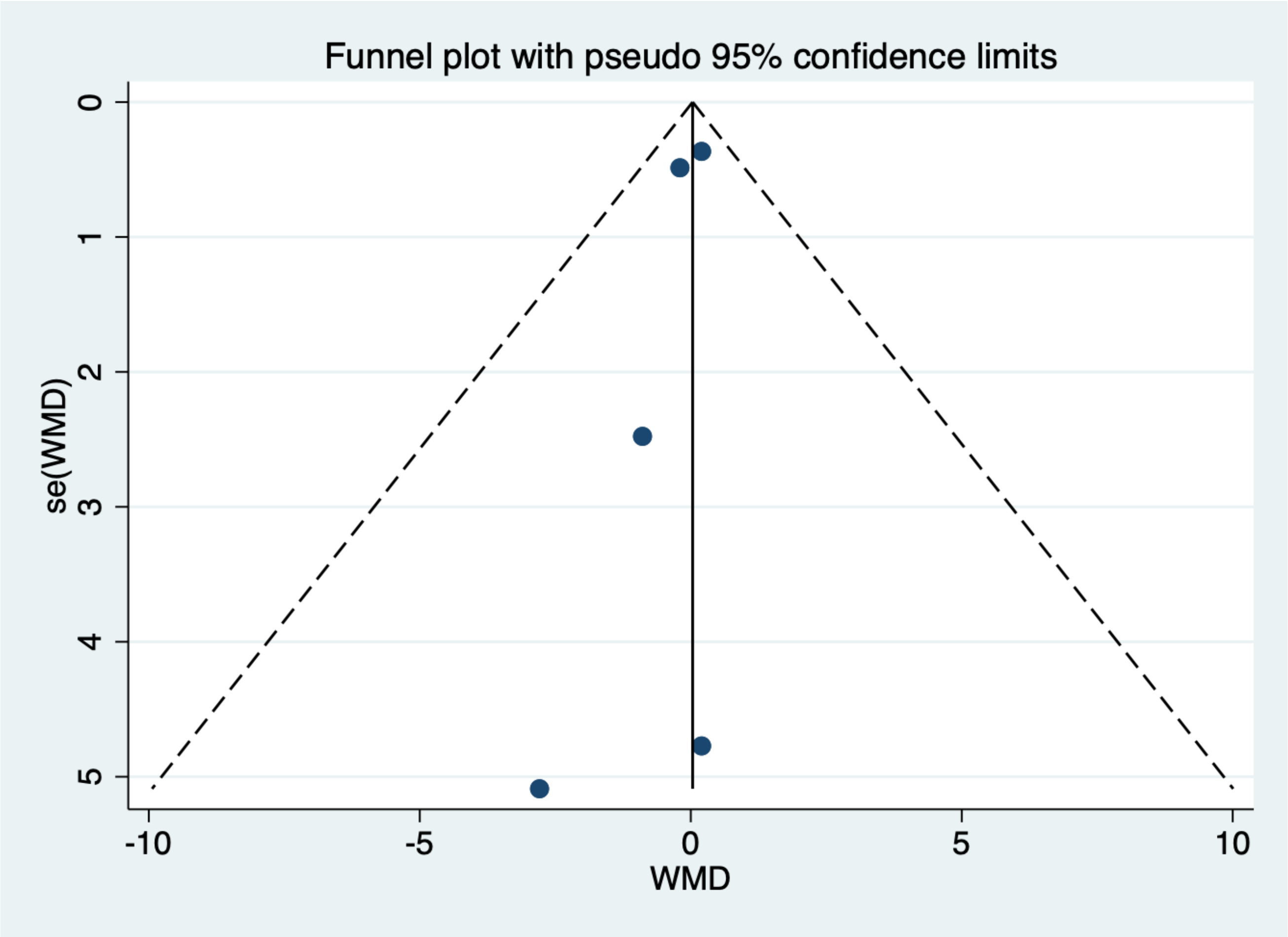

Supplementary Figure 3. The funnel plot of body mass.

4 The quality of the evidence (GRADE)

Supplementary Table 3.

Quality assessment							NO of patients		Effect	Certainty
**No of studies**	**Design**	**Risk of bias**	**Inconsistency**	**Indirectness**	**Imprecision**	**Other considerations**	**ES intervention**	**Comparison**	**Absolute**	
Lean mass										
4	randomised trials	not serious	not serious	not serious	very serious[a]	none	46	48	MD 0.52kg higher (0.13 higher to 0.91 higher)	⊕⊕◯◯ Low
Body fat										
5	randomised trials	not serious	not serious	not serious	serious[a]	none	77	64	MD -0.04% lower (-0.73 lower to 0.65 higher)	⊕⊕⊕◯ Moderate
Body mass										
5	randomised trials	not serious	not serious	not serious	serious[a]	none	103	90	MD 0.03kg (-0.53 lower to 0.60 higher)	⊕⊕⊕◯ Moderate

*GRADE: Quality of Clinical Evidence and Recommendation Levels:
High: The research team has strong confidence in the estimated effect size;
Moderate: The research team has moderate confidence in the estimated effect size;
Low: The research team has limited confidence in the estimated effect size;
Very low: The research team has very limited confidence in the estimated effect size.

*Explanations:
a. Serious imprecision due to small sample size and wide confidence intervals.
